# Supplementary material for: Quantum-chemical insights from deep tensor neural networks
Source: Nat Commun. 2017 Jan 9;8:13890. doi: 10.1038/ncomms13890 (PMC5228054; doi:10.1038/ncomms13890)
Supplement: Supplementary Information — Supplementary Figures, Supplementary Tables, Supplementary Discussion and Supplementary Methods [file ncomms13890-s1.pdf]

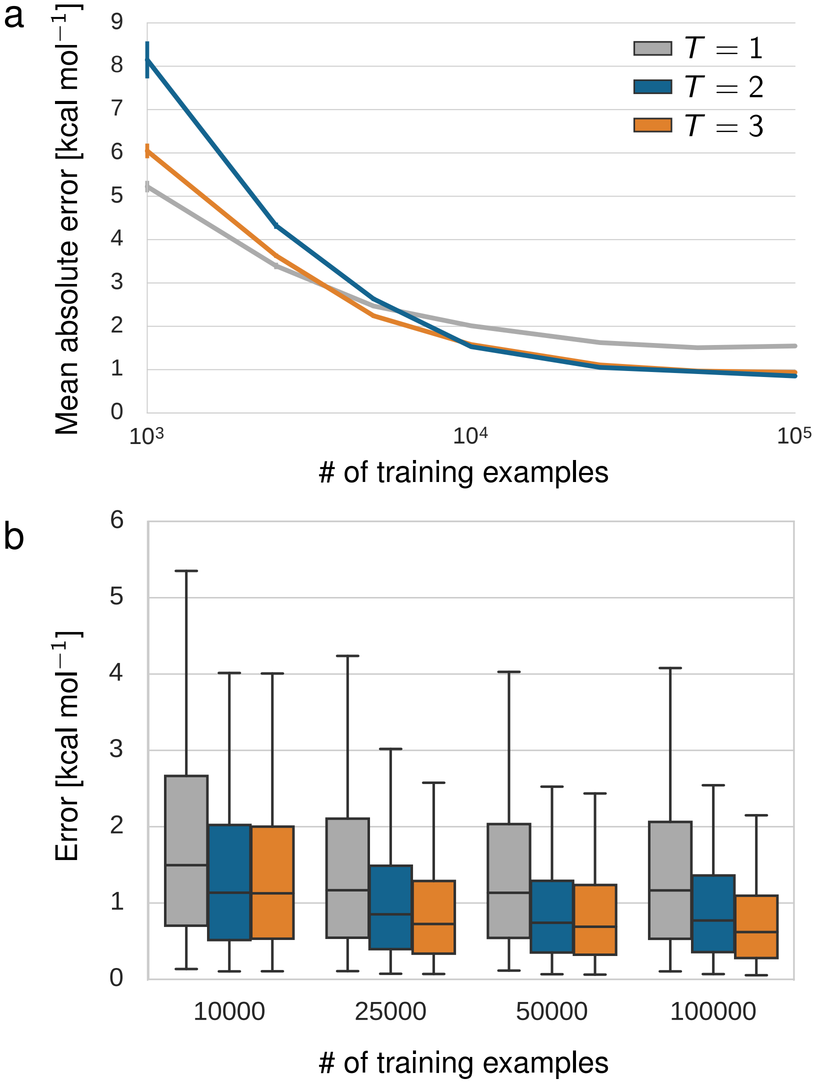

Supplementary Figure 1: **Chemical compound space. Errors depending on the size of the training set for models with $T = 1, 2, 3$ interaction passes trained on GDB-9.** (a) Mean absolute error of neural networks depending on the number of training examples. Error bars correspond to standard errors over five repetitions. For more than 5k examples, the error bars vanish due to standard errors below 0.05 kcal mol^{-1}. (b) Error distribution for models trained on 10k, 25k, 50k and 100k training examples. The box spans between the 25% and 75% quantiles, while the whiskers mark the 5% and 95% quantiles.

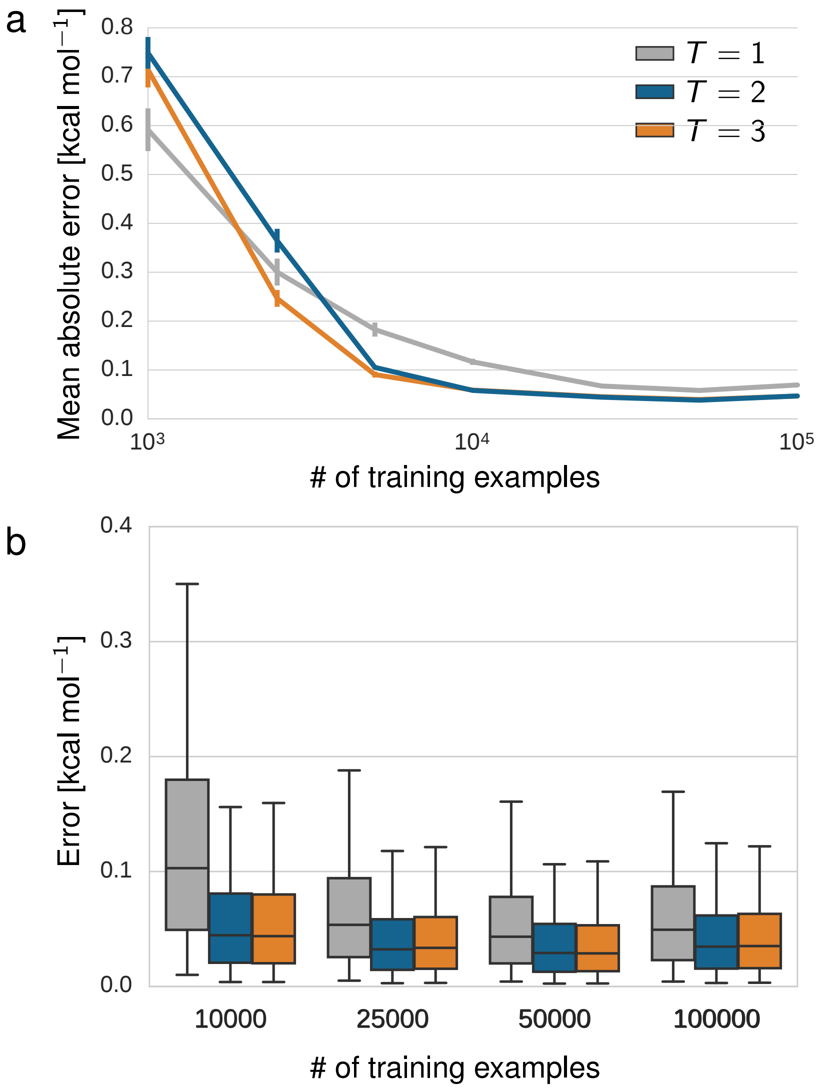

Supplementary Figure 2: **Molecular dynamics. Errors depending on the size of the training set for models with $T = 1, 2, 3$ interaction passes trained on the Benzene data set.** (a) Mean absolute error of neural networks depending on the number of training examples. Error bars correspond to standard errors over five repetitions. For more than 10k examples, the error bars vanish due to standard errors below 0.01 kcal mol^{-1}. (b) Error distribution for models trained on 10k, 25k, 50k and 100k training examples. The box spans between the 25% and 75% quantiles, while the whiskers mark the 5% and 95% quantiles.

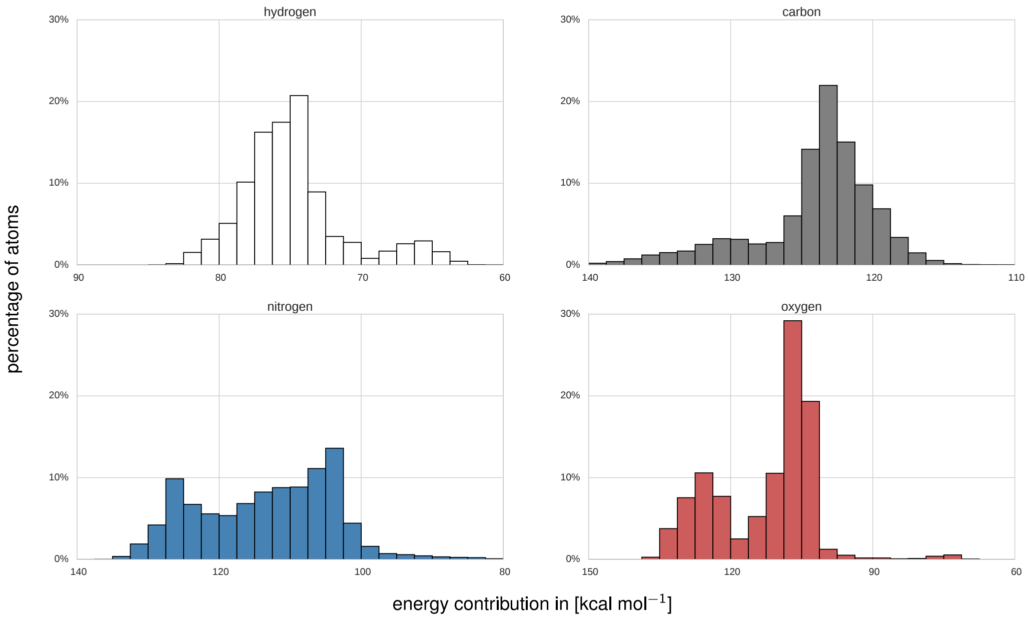

Supplementary Figure 3: **Distribution of atomic energy contributions E_i in the GDB-9 data set.** The energy contributions were predicted using the GDB-9 model with two interaction passes trained on 50k reference calculations.

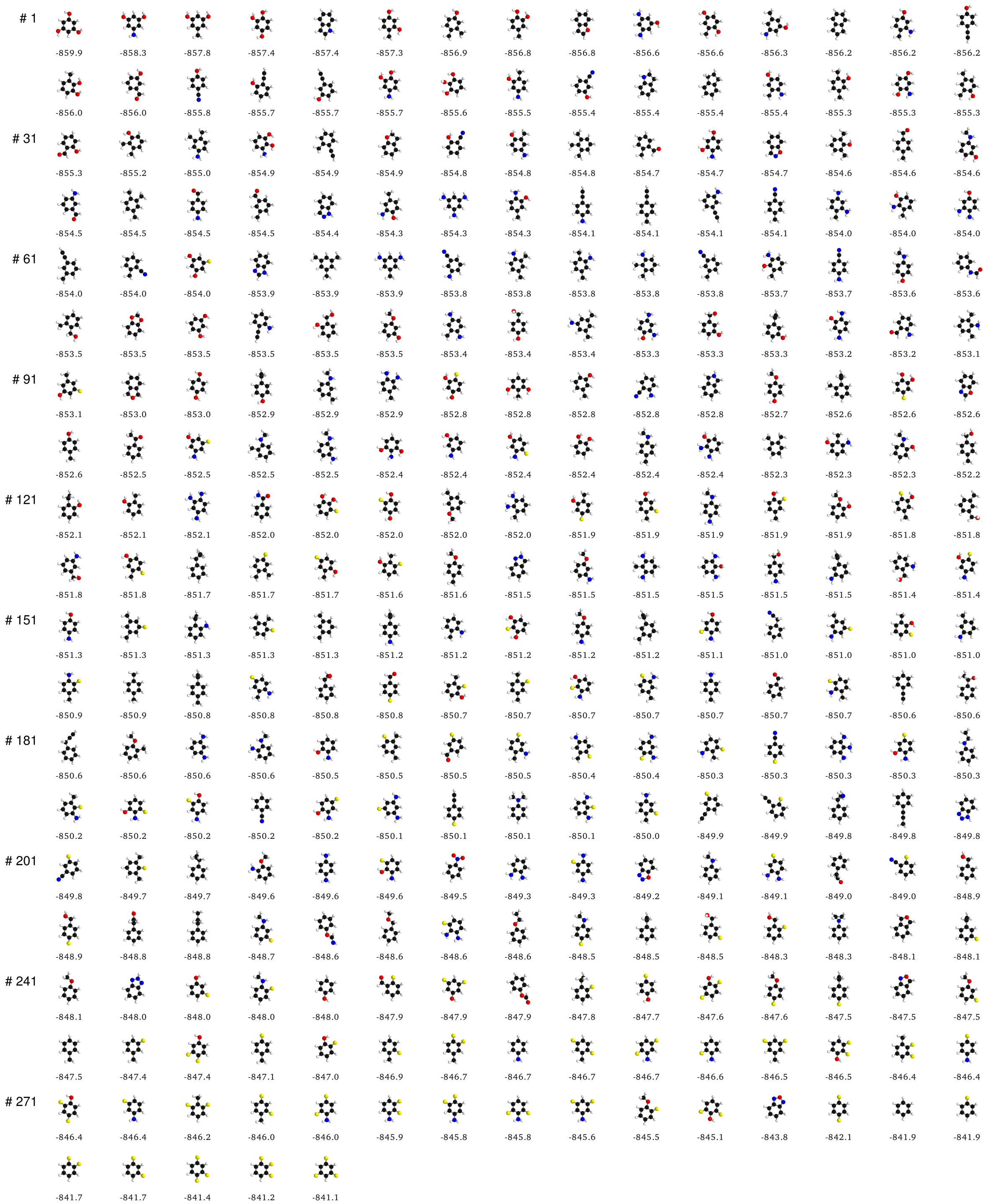

Supplementary Figure 4: **List of 6-membered carbon rings ordered by the sum of energy contributions of the ring atoms.** The energy contributions were predicted using the GDB-9 model with three interaction passes trained on 50k reference calculations. Energy contributions are given in kcal mol^{-1}.

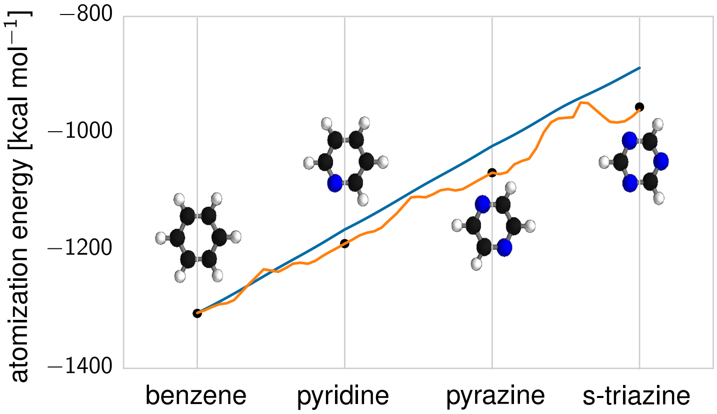

Supplementary Figure 5: **An alchemical path of the DTNN trained on 50k GDB-9 reference calculations with $T = 2$.** The DTNN model is able to smoothly create, remove and move atoms as well as continuously change their element-specific characteristics. A path leading from benzene to s-triazine was computed by only changing, removing and changing types of atoms (blue). In the second path (orange), atoms were also moved to the new equilibrium positions. The black dots mark the energy of DFT reference calculations.

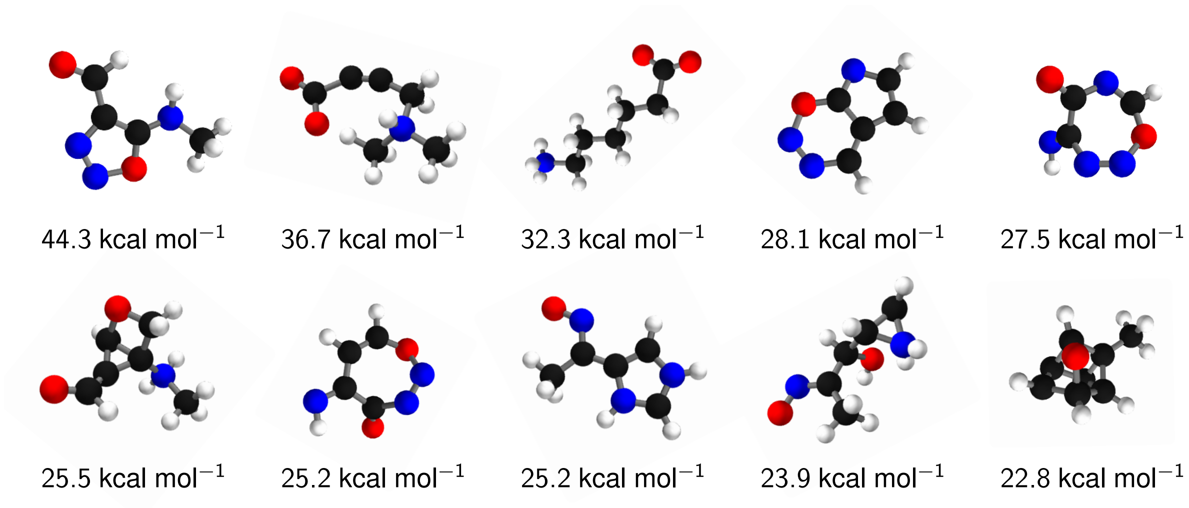

Supplementary Figure 6: **Top-10 largest prediction errors on the GDB-9 model with two interaction passes trained on 50k reference calculations.**

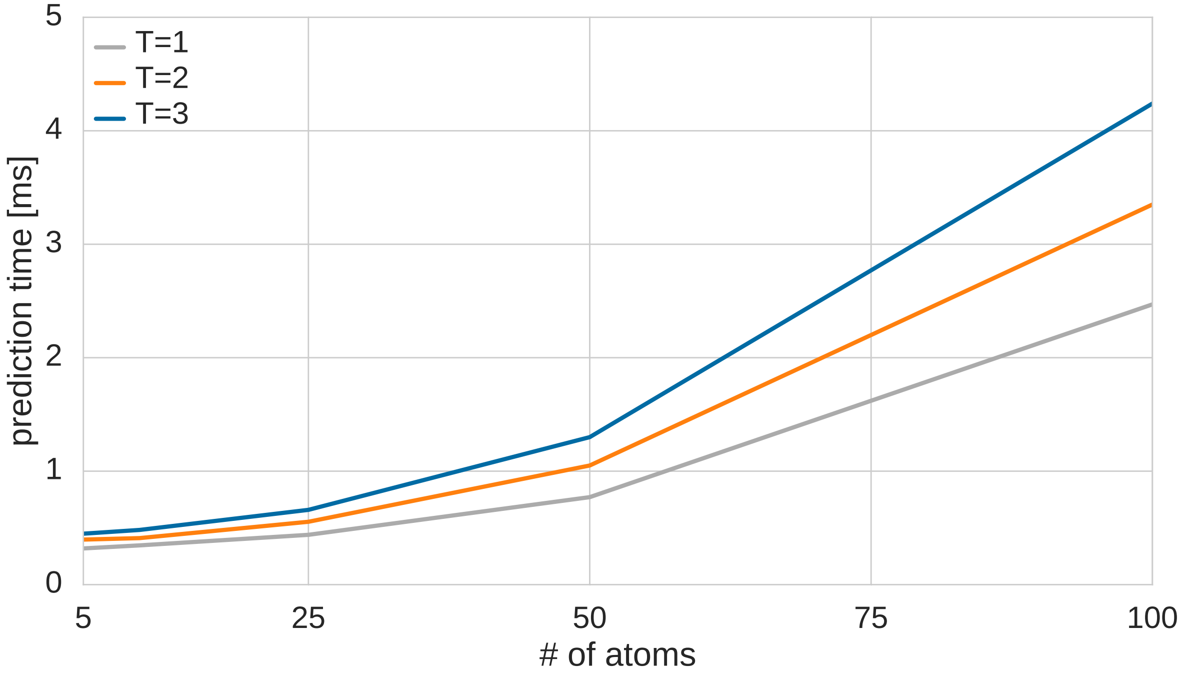

Supplementary Figure 7: **Prediction time needed for a molecule depending on the number of atoms and number of interaction passes T of the employed DTNN.** All predictions were computed on an NVIDIA Tesla K40 GPU.

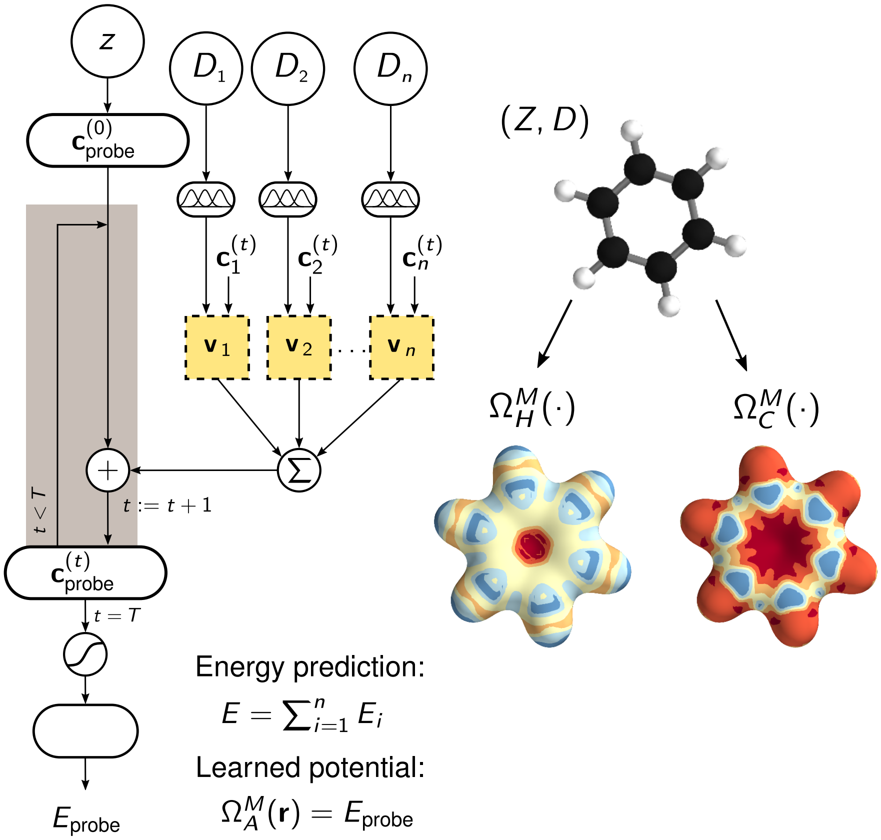

Supplementary Figure 8: **Illustration of how the surface plots are obtained from a trained network as shown in Fig. 1.** The deep network can be interpreted as representing a local potential $\Omega_A^M(\mathbf{r})$ created by the atoms of the molecule. Putting a probe atom A with nuclear charge z at a position $\mathbf{r}$ described by the distances to the atoms of the molecule $d_1, \ldots, d_n$ yields an energy E_{probe}.

Supplementary Table 1: **Errors of neural networks with up to three interaction passes for various data sets and numbers of reference calculations used in training**

Data set	# training data	T=1		T=2		T=3	
		MAE [kcal mol^{-1}]	RMSE [kcal mol^{-1}]	MAE [kcal mol^{-1}]	RMSE [kcal mol^{-1}]	MAE [kcal mol^{-1}]	RMSE [kcal mol^{-1}]
GDB-7 [a]	5768	1.28 ± 0.04	1.99 ± 0.14	**1.04 ± 0.02**	**1.43 ± 0.02**	**1.04 ± 0.01**	1.45 ± 0.01
GDB-9 [b]	25k	1.61 ± 0.02	2.31 ± 0.02	1.09 ± 0.01	1.62 ± 0.02	**1.04 ± 0.02**	**1.53 ± 0.02**
	50k	1.49 ± 0.02	2.14 ± 0.03	0.96 ± 0.01	**1.37 ± 0.03**	**0.94 ± 0.01**	**1.37 ± 0.01**
	100k	1.54 ± 0.03	2.17 ± 0.04	0.93 ± 0.02	1.33 ± 0.03	**0.84 ± 0.02**	**1.21 ± 0.02**
Benzene [b]	25k	0.07 ± 0.00	0.10 ± 0.00	0.05 ± 0.00	**0.06 ± 0.00**	**0.04 ± 0.00**	**0.06 ± 0.00**
	50k	0.06 ± 0.00	0.08 ± 0.00	**0.04 ± 0.00**	**0.05 ± 0.00**	**0.04 ± 0.00**	**0.05 ± 0.00**
	100k	0.07 ± 0.00	0.10 ± 0.00	**0.05 ± 0.00**	**0.06 ± 0.00**	**0.05 ± 0.00**	**0.06 ± 0.00**
Toluene [b]	25k	0.48 ± 0.01	0.63 ± 0.01	**0.20 ± 0.00**	**0.28 ± 0.00**	0.23 ± 0.00	0.31 ± 0.01
	50k	0.44 ± 0.00	0.59 ± 0.01	**0.18 ± 0.00**	**0.24 ± 0.00**	**0.18 ± 0.00**	**0.24 ± 0.00**
	100k	0.42 ± 0.01	0.56 ± 0.01	**0.16 ± 0.00**	**0.21 ± 0.00**	0.17 ± 0.00	0.22 ± 0.00
Malonaldehyde [b]	25k	0.54 ± 0.00	0.74 ± 0.00	**0.23 ± 0.00**	0.34 ± 0.00	**0.23 ± 0.00**	**0.33 ± 0.00**
	50k	0.49 ± 0.01	0.68 ± 0.01	0.20 ± 0.00	0.28 ± 0.00	**0.19 ± 0.00**	**0.27 ± 0.00**
	100k	0.51 ± 0.01	0.70 ± 0.01	0.18 ± 0.00	0.25 ± 0.00	**0.17 ± 0.00**	**0.24 ± 0.00**
Salicylic acid [b]	25k	0.80 ± 0.02	1.05 ± 0.03	**0.54 ± 0.02**	**0.72 ± 0.03**	0.79 ± 0.02	1.03 ± 0.03
	50k	0.73 ± 0.01	0.94 ± 0.01	**0.41 ± 0.00**	**0.54 ± 0.00**	0.50 ± 0.01	0.65 ± 0.01
	100k	0.67 ± 0.01	0.88 ± 0.01	**0.39 ± 0.01**	**0.51 ± 0.01**	0.42 ± 0.01	0.54 ± 0.01

Mean absolute errors (MAE), root mean squared errors (RMSE) as well as respective standard errors of the mean are printed. The maximum error over all folds is given. Best results are printed in bold.
[a] 10% of the reference calculations are used as validation set for early stopping.
[b] 1k reference calculations are used as validation set for early stopping.

Supplementary Table 2: **Training duration for the presented neural networks with up to three interaction passes** ($T = 1, 2, 3$)

Data set	# training examples	$T = 1$	$T = 2$	$T = 3$
GDB-7	5768	6	7	8
GDB-9	25k	28	35	42
	50k	55	71	82
	100k	110	139	162
Benzene	25k	21	27	32
	50k	44	53	61
	100k	84	104	121
Toluene	25k	24	27	32
	50k	45	55	64
	100k	88	108	127
Malonaldehyde	25k	21	25	29
	50k	41	52	59
	100k	85	106	117
Salicylic acid	25k	22	31	32
	50k	44	54	65
	100k	91	109	125

All durations in hours. All models were trained using stochastic gradient descent with momentum for 3.000 epochs on an NVIDIA Tesla K40 GPU.

Supplementary Discussion

Performance depending on number of reference calculations and interaction passes

Supplementary Figs. **1** and **2** show the dependence of the performance on the number of training examples for the benzene MD data set and GDB-9, respectively. In both learning curves (a), an increase from 1.000 to 10.000 training examples reduces the error drastically while another increase to 100.000 examples yields comparatively small improvement. The error distributions (b) show that models with two and three interaction passes trained on at least 25.000 GDB-9 references calculations predict 95% of the unknown molecules with an error of 3.0 kcal mol^{-1} or lower. Correspondingly, the same models trained on 25.000 or more MD reference calculations of benzene predict 95% of the unknown benzene configurations with a maximum error lower than 1.3 kcal mol^{-1}.

Beyond a certain number of reference calculations, the models with one interaction pass perform significantly worse in all theses respects. Thus, multiple interaction passes indeed enrich the learned feature representation as demonstrated by the increased predictability of previously unseen molecules.

Relation to convolutional neural networks

In a convolution layer, local filters are applied to local environments, e.g., image patches, extracting features relevant to the classification task. Similarly, local correlations of atoms may be exploited in a chemistry setting. The atom interaction in our model can indeed be regarded as a non-linear generalization of a convolution. In contrast to images however, atoms of molecules are not arranged on a grid. Therefore, the convolution kernels need to be continuous. We define a function $C^t : R^3 \rightarrow R^B$ yielding $\mathbf{c}_i^t = C^t(\mathbf{r}_i)$ at the atom positions. Now, we can rewrite the interactions as

$$C^{t+1}(\mathbf{r}_i)_k = C^t(\mathbf{r}_i)_k + \sum_{j \neq i} h(f(\mathbf{r}_j)_k g(\|\mathbf{r}_j - \mathbf{r}_i\|)_k), \tag{1}$$

with

$$f(\mathbf{r}_j) = W^{\mathrm{cf}} C^t(\mathbf{r}_j) + \mathbf{b}^{\mathrm{f}_1}, \tag{2}$$

$$g(d_{ij}) = W^{\mathrm{df}} \hat{\mathbf{d}}_{\mathbf{ij}} + \mathbf{b}^{\mathrm{f}_2}, \tag{3}$$

$$h(x) = \tanh(W^{\mathrm{fc}} \mathbf{x}). \tag{4}$$

For h being the identity, the sum is equivalent to a discrete convolution of f and g.

Supplementary Methods

Computing an alchemical path with the DTNN

The alchemical paths in Supplementary Fig. 5 were generated by gradually moving the atoms as well as interpolating between the initial coefficient vectors for changes of atom types. Given two nuclear charges A, B, the coefficient vector for any charge $Z_i = \alpha_i A + (1 - \alpha) B$ with $0 \leq \alpha \leq 1$ is given by

$$\mathbf{c}_{Z_i} = \alpha_i \mathbf{c}_A + (1 - \alpha_i) \mathbf{c}_B. \tag{5}$$

Similarly, in order to add or remove atoms, we introduce fading factors $\beta_1, \ldots, \beta_n \in [0, 1]$ for each atom. This way, influences on other atoms

$$\mathbf{c}_i^{(t+1)} = \mathbf{c}_i^{(t)} + \sum_{j \neq i} \beta_j v(\mathbf{c}_j^{(t)}, D_{ij}) \tag{6}$$

as well as energy contributions to the molecular energy $E = \sum_{i=1}^{n} \beta_i E_i$ can be faded out.
